# Energy and Protein in Critically Ill Patients with AKI: A Prospective, Multicenter Observational Study Using Indirect Calorimetry and Protein Catabolic Rate

**DOI:** 10.3390/nu9080802

**Published:** 2017-07-26

**Authors:** Alice Sabatino, Miriam Theilla, Moran Hellerman, Pierre Singer, Umberto Maggiore, Maria Barbagallo, Giuseppe Regolisti, Enrico Fiaccadori

**Affiliations:** 1 Acute and Chronic Renal Failure Unit, Department of Clinical and Experimental Medicine Renal ICU, Parma University Hospital, Parma 43126, Italy; alice.sabatino86@gmail.com (A.S.); giuregolisti@gmail.com (G.R.); 2 Department of General Intensive Care and Institute for Nutrition Research, Rabin Medical Center, Beilinson Hospital, Petah Tikva, Affiliated with the Sackler School of Medicine, Tel Aviv University, Tel Aviv 49100, Israel; miriamt@clalit.org.il (M.T.); moranhe@clalit.org.il (M.H.); psinger@clalit.org.il (P.S.); 3 Kidney-Pancreas Transplant Unit, Parma University Hospital, Parma 43126, Italy; umberto_maggiore@hotmail.com; 4 Surgical ICU, Anesthesia, Intensive Care and Pain Therapy, Parma University Hospital, Parma 43126, Italy; mbarbagallo@parmanesthesia.com

**Keywords:** acute kidney injury, artificial nutrition, indirect calorimetry, overfeeding, protein catabolic rate, underfeeding

## Abstract

The optimal nutritional support in Acute Kidney Injury (AKI) still remains an open issue. The present study was aimed at evaluating the validity of conventional predictive formulas for the calculation of both energy expenditure and protein needs in critically ill patients with AKI. A prospective, multicenter, observational study was conducted on adult patients hospitalized with AKI in three different intensive care units (ICU). Nutrient needs were estimated by different methods: the Guidelines of the European Society of Parenteral and Enteral Nutrition (ESPEN) for both calories and proteins, the Harris-Benedict equation, the Penn-State and Faisy-Fagon equations for energy. Actual energy and protein needs were repeatedly measured by indirect calorimetry (IC) and protein catabolic rate (PCR) until oral nutrition start, hospital discharge or renal function recovery. Forty-two patients with AKI were enrolled, with 130 IC and 123 PCR measurements obtained over 654 days of artificial nutrition. No predictive formula was precise enough, and Bland-Altman plots wide limits of agreement for all equations highlight the potential to under- or overfeed individual patients. Conventional predictive formulas may frequently lead to incorrect energy and protein need estimation. In critically ill patients with AKI an increased risk for under- or overfeeding is likely when nutrient needs are estimated instead of measured.

## 1. Introduction

Acute Kidney Injury (AKI) is now considered a major clinical health problem [1,2]. In fact, its incidence is increasing, especially among ICU patients, and the syndrome still remains associated with highly negative outcomes [3,4]. Severe malnutrition has been documented in up to 40% of patients with AKI, and is associated with a further increase in the mortality risk and complications [5].

Accurate determination of protein and energy needs is obviously important in this clinical setting, because both over- and underfeeding may occur and are associated with poor outcomes [6,7]. In particular, significant underfeeding has been recently documented in a recent re-analysis of data from a large cohort of patients with AKI from the RENAL study, whose average daily calorie and protein intake was respectively 867 kcal and 34.8 g [8].

The gold standard for measuring individual caloric needs is represented by indirect calorimetry (IC), a noninvasive method allowing resting energy expenditure (REE) assessment based on oxygen consumption and carbon dioxide production measurements in the exhaled air [9]. In critically ill patients, REE measured by IC is in general considered for nutritional prescription. However, during the very early phase (2–3 days) of an acute illness the body produces enough amount of energy to cover most of energy needs, increasing the risk for early overfeeding [9]. Regarding proteins, the daily protein catabolic rate (PCR as g/24 h) reflects the amount of protein undergoing catabolism and could be considered the target for protein prescription. PCR can be derived from cumulative nitrogen excretion and urea kinetics, in the case of Renal Replacement Therapy (RRT), and can also be expressed as normalized PCR (nPCR, as g/kg of BW/24 h) [10]. Unfortunately, IC measurements and nPCR are not widely used in daily ICU routine [9], due to time constraints, reduced equipment availability, staffing scarcity, and costs [9]. On the other hand, the important limitations and the lack of accuracy of conventional predictive equations for EE estimation in critically ill are well known [11,12,13,14,15]. Moreover, data on their validity when applied to patients with AKI are scanty [16]. Regarding proteins, while PCR of patients on RRT has been investigated in the past by urea kinetic methods [10,17,18,19,20,21], no data are currently available on PCR in patients with AKI not on RRT.

Thus, the purpose of our study in patients with AKI with or without RRT need was:

(a) to evaluate the validity of conventional predictive formulas and equations for the calculation of energy expenditure and protein needs, by using IC and the nPCR as gold standards;
(b) to compare prescribed and actually received nutrients with estimated and measured needs.

## 2. Materials and Methods

### 2.1. Patients

This prospective observational study was conducted at the Renal and the Surgical ICUs of the Parma University Hospital (Parma, Italy) and at the General ICU of the Rabin Medical Center Beilinson Hospital (Tel Aviv, Israel) from October 2014 to November 2015. The procedures followed were in accordance with the Helsinki Declaration. The local Institutional Review Boards approved the study. Written informed consent was obtained from the patients, or from an authorized next of kin/legal representative.

Adult patients with stage 3 AKI [22], were included, with or without mechanical ventilation need, on exclusive artificial nutrition (enteral and/or parenteral) at the time of the first IC, and expected to have an ICU stay of at least 3 days. Exclusion criteria were based both on specific comorbidities and on the presence of factors that could interfere with REE measurements accuracy [9]. Patients with end-stage renal disease (ESRD) on chronic dialysis or peritoneal dialysis, severe liver failure, pregnancy, head trauma and burns were also excluded.

### 2.2. Data Collection and Calculations

General demographic and clinical variables were collected. Daily nutritional prescriptions and intakes were recorded up to the end of ICU stay or the beginning of oral feeding. Energy needs were estimated according to ESPEN guidelines for critically ill patients with AKI (25 total kcal/kg body weight (BW)) [23,24], and from the calculated Resting Energy Expenditure (REE) values obtained by 3 different equations widely used in the ICU setting: the Faisy-Fagon [12] and the Penn-State equations [13] for mechanically ventilated patients, and the Harris-Benedict equation [25]. The detailed equations are described in the Supplementary Table S1. Protein needs were estimated according to ESPEN guidelines for critically ill patients with AKI (1 g/kg/day for patients not on RRT and 1.7 g/kg/day for patients undergoing RRT) [23,24]. Actual energy needs were measured by IC every 48 h during the first week of study, and then once a week until beginning of oral feeding, recovery of renal function or the end of ICU stay. Normalized protein catabolic rate was calculated as previously described in detail [10,26].

### 2.3. Indirect Calorimetry Instrumentation and Measurements

The portable Vmax^TM^ Encore Metabolic Cart 29n (SensorMedics Italia srl, Milano, Italy) or the Deltatrac II (Datex-Omeda, GE, Helsinki, Finland) were used for IC measurements. Before each measurement, flow sensor and gas calibration were performed with a fixed gas concentration. The IC measurements were performed in the morning, at a non-fasting steady state, at least 2 h after any intervention for a 30 min period. Reference weight was measured by electronic bed scales. In patients on RRT, IC measurements were performed in the interdialytic period, at least two hours after the end of dialysis. In addition, on the day of indirect calorimetry measurement additional variables that could impact on metabolism were collected (i.e., body temperature, vasoactive drugs, sedatives).

### 2.4. Artificial Nutrition

No changes were made in the routine of nutritional support for the patients, as per the protocol of each ICU. Enteral nutrition was the first choice if no major contraindication was present. Parenteral nutrition was added in case estimated needs were not reached in 3 days.

### 2.5. Reference Body Weight

As per protocol of the ICUs involved, reference BW used for the prediction of energy and protein needs was preferably measured using electronic bed scales. In the case of patients undergoing RRT, the BW measured at the end of RRT was used, while for patients not on RRT the most recent BW before IC measurement was considered for calculations. BW at admission was used only when no data on recent BW were available.

### 2.6. Statistical Analysis

Results are expressed as mean and standard deviation for continuous variables with normal distribution, or median and range for non-parametric data, and as frequencies for categorical variables. The pairwise differences between means of the total prescribed and delivered energy and proteins were tested by paired *t* test or Wilcoxon for non-parametric variables. Difference between predictive equations, prescribed and delivered energy/proteins and IC/PCR values were tested with one-way ANOVA; differences between the means of each equation with IC measurements were tested by Wilcoxon for non-parametric variables. Normality was assessed with the Kolmogorov-Smirnov test. Agreement between methods was assessed by the Bland-Altman approach [27]. To consider an equation accurate, limits of agreement should be ±10% of REE or ±250 Kcal [16,28,29,30]. Patients who achieved intakes within 90–110% of calculated targets (±10% of REE) were considered as “adequately fed”. To analyze precision, we considered that if the majority (>50%) of individual differences were outside the defined range considered as “adequately fed”, the method was considered imprecise and clinically unacceptable. A linear mixed model was used to compare REE measured by indirect calorimetry performed at least twice in the same patients within 7 days since ICU admission. Data analysis was performed using GraphPad Prism version 6.00 for Windows (GraphPad Software, La Jolla, San Diego, CA, USA) and SPSS statistics for Windows (Version 23, IBM Corp., Armonk, NY, USA).

## 3. Results

Demographic and clinical data are described in Table 1. We enrolled 42 critically ill patients, 72% (31/42) were male, mean age was 67 (±15) years, BMI at admission 29 (±9.4) kg/m^2^ (Table 1). Length of ICU stay (LOS) was 17 (5–99) days, hospital LOS was 41 (5–137) days; 24% (10/42) of patients died during ICU stay, while the in-hospital mortality was 38% (16/42); patients were mechanically ventilated for 12 (1–84) days. Forty-five percent (19/42) of patients needed RRT; Sustained-low efficiency dialysis (SLED, 8–12h) was performed in 14/19 (74%) patients, continuous veno-venous hemofiltration (CVVH) in 2/19 (20%), conventional hemodialysis in 3/19 (16%). During the 130 IC measurements, the number of RRT treatments in the last 24 h before measurements corresponded to 38, and SLED was the most used modality (32/38, 84%) (Table 1). Median duration of RRT was 9 (1–86) days; 6 out 19 patients (32%) were still on RRT at ICU discharge/death. Clinical data on the day of IC measurement are described in Table 2. The average maximum temperature achieved in the last 24 h before IC measurement was 37 (±0.9) °C, and the majority of patients (57%) were using sedative drugs.

### 3.1. Nutrition

There were 654 days of artificial nutrition (Table 3), 45% (294/654) on EN alone and 24% (156/649) on enteral plus parenteral nutrition. Average energy and protein prescribed were respectively 1551 ± 644 kcal and 70.5 ± 38.2 g, while energy and protein actually delivered were 1408 ± 651 kcal and 63.4 ± 35.3 g (*p* < 0.0001 for both comparisons).

#### 3.1.1. Indirect Calorimetry and Predictive Equations

**Figure 1:**
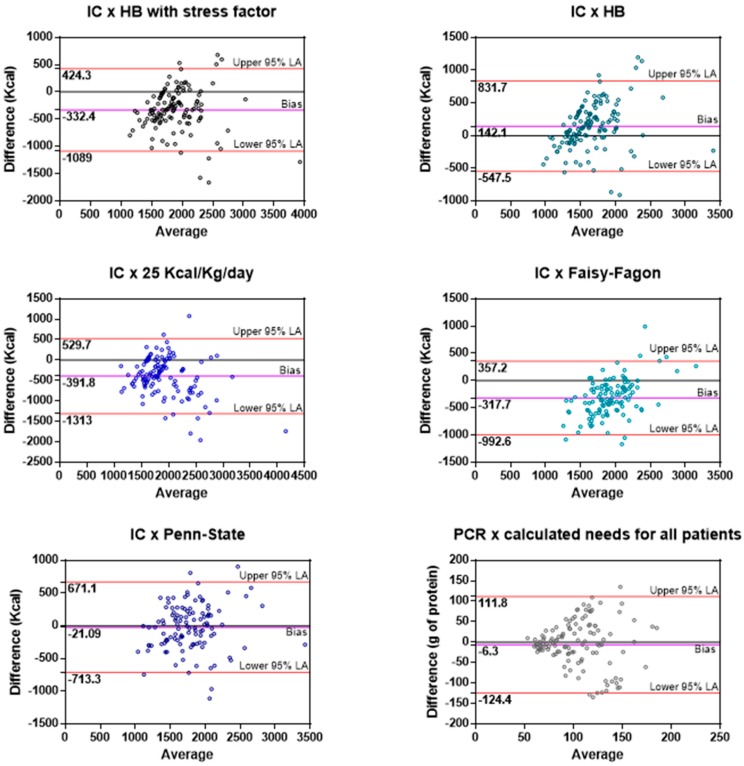
Bland-Altman plots of the agreements between indirect calorimetry or protein catabolic rate and predictive equations.

Table 4 shows the comparison between IC measurements, predictive equations and prescribed/delivered calories. There were 130 IC measurements over 654 days of nutrition. Mean daily energy needs by IC were 1724 kcal ± 431 kcal (1771 ± 431 kcal in ventilated patients). In general, average energy needs measured by IC were significantly higher than both the prescribed and delivered nutrient amounts (IC 1724 kcal ± 431; prescribed 1575 ± 672; received 1439 ± 680, *p* < 0.0001). No difference was observed between measured energy expenditure in patients on RRT vs. those not on RRT (*p* = 0.9184). The predictive equation better estimating energy needs was the Penn-State equation. Bland-Altman analysis was used for a visual appraisal of agreements between predictive equations and IC (Figure 1). The wide limits of agreement in each case highlight the potential for under- or overfeeding in individual patients that might arise when these predictive equations are used. The percentage of estimates, calculated using the different equations, that fall into the “adequate”, under- and overfeeding categories are shown in Table 5. Considering that AKI can cause fluid overload and that we were using the actual body weight of patients for the REE estimations, we decided to repeat the analysis considering only IC measurements and equations that were not performed during a period of fluid overload. Nineteen patients (45%) presented fluid overload at some point during recovery, with a mean delta fluid overload (calculated with the maximum and minimum body weight during ICU stay) of 8 ± 5.4 L. In addition, we excluded 5 patients for whom the admission body weight was not available. Days on fluid overload corresponded to 49 IC measurements. Excluding these 49 measurements, we analyzed the remaining 81 measurements (72 measurements for ventilated patients). The results were unchanged (Table 6). For what concern the precision of the equations, no equation had the majority of estimates into the adequate category. Patients were submitted to 3 (1–11) IC and 2 (1–15) PCR measurements during ICU stay. IC measurements were performed at least twice within 7 days since ICU admission in 25 (92.6%) of the 27 patients that had more than 1 measurement. Ten patients (37%) had 2 measurements, 7 patients (25.9%) 3, 7 patients (25.9%) 4, and 1 patient (3.7%) had 5 measurements within this time frame. No significant differences were detected in absolute values between the first and the last IC measurements (patients with 2 measurements (mean ± SEM) 1738 ± 121 vs. 1911 ± 156 kcal/day, *p* = 0.11; patients with 3 measurements 1709 ± 107 vs. 1733 ± 199 kcal/day, *p* = 0.18; patients with 4 measurements 1570 ± 106 vs. 1819 ± 211 kcal/day, *p* = 0.36). Nineteen patients (19/27, 70%) had a second IC measurement within 48 h from baseline; even despite the mean difference was not statistically significant (1635 ± 350 vs. 1705 ± 482, *p* = 0.42), in 68% of those patients (13/19) the difference between measurements was ≥+10% or ≤−10%, which is clinically relevant.

FF, Faisy-Fagon (ventilated patients); HB, Harris-Benedict; IC, indirect calorimetry; PCR, protein catabolic rate; Penn, Penn-State (ventilated patients).

#### 3.1.2. Protein Catabolic Rate and Recommended Formulas

In general, PCR values were higher than the prescribed and the actually received daily protein intakes (PCR 102.1 *g* ± 38.7, prescribed 76.5 ± 37.3, received 69.2 ± 35.3, *p* < 0.0001) (Table 4). Patients on RRT were more likely to be prescribed adequate protein amounts (PCR vs. prescribed proteins *p* = 0.3205); however, these patients actually received significantly less proteins than PCR (*p* = 0.02). Nevertheless, estimated protein needs were much higher than PCR (154.0 ± 51.8 vs. 89.5 *g* ± 29.3, *p* < 0.0001).

The Bland-Altman plot underscores the wide limits of agreement between PCR and calculated protein needs (Figure 1), leading to an increased risk of insufficient or excess protein intake.

In 46% of estimates (56/123), protein needs were overestimated. Only in 6 estimations (15%) in the RRT group the estimated intake was able to correctly predict protein needs (within ±10% of measured PCR). In the AKI subgroup of patients not on RRT protein needs were frequently underestimated (56%, 47/84). Over 68% of the artificial nutrition days patients received less than 90% of PCR. Particularly, in patients not on RRT protein intake was lower than PCR over 75% of the time, while patients on RRT were underfed over 54% of the time. Adequate protein intake was given only in 13% of the measured artificial nutrition days. We also performed these analysis again considering only measurements not on fluid overload (*n* = 69). The results were unchanged (PCR 94.1 ± 43.8 versus Equation 90.2 ± 35.7, *p* = 0.61; PCR no RRT 97.8 ± 46.7 vs. Equation 75.4 ± 16.2, *p* = 0.0023; and PCR RRT 80.9 ± 28.5 vs. 143.6 ± 36.2, *p* = 0.0003). The Bland-Altman analysis continued to report wide limits of agreement (bias 3.89 ± 56.3, 95% Limits of agreement: −106.5 to 114.3).

## 4. Discussion

Our study suggests that in critically ill patients with AKI none of the conventional equations and formulas used to predict REE is in good agreement with actual EE measured by IC, nor protein needs were correctly estimated. Moreover, actual nutrients intakes were significantly lower as compared to both the prescribed and the measured needs. 

The most ancient and widely used equation is the Harris-Benedict, developed from healthy caucasic subjects in the beginning of the 20th century [25]. In the present study, this equation was able to correctly predict EE over only 38% of time. In a very recent study on AKI patients, the same equation was the least precise, with only about 18% of cases predicting EE within 10% of the measured EE [16]. In a systematic review of the literature on the accuracy of predictive equations for critically ill patients, the Harris-Benedict equation underestimated EE and the use of correction factors was suggested to adjust EE to the current individual situation [13]. In the present study, the widely applied stress factor of 1.3 was used [31]. The use of this factor unfortunately worsened the precision of the equation when applied to patients with AKI, with only 26% of estimates between 90–110% of EE measured by IC and 69% of estimates >110% of IC. An important factor likely to have increased the estimation error could be represented by the body weight value used for the calculations. In the case of patients undergoing RRT, the BW at the end of RRT was used, while for patients not on RRT the most recent BW before the IC measurement was considered for calculations. Other studies in critically ill patients also showed a low grade of agreement between Harris-Benedict equation—with and without injury factor correction—and EE measured by IC [11,12,15,16,32,33,34,35]. In addition, review studies do not recommend the use of Harris-Benedict equation, with or without injury factor, for critically ill patients [13,36].

Guidelines on renal and ICU patients [23,24,37] recommend 20–25 kcal/kg/day during the acute phase of the disease, and 25–30 kcal/kg/day during recovery. However, in many cases it is not mentioned if the actual, pre-admission or ideal BW should be considered. In the present study, the use of 25 kcal/kg/day calculation overestimated EE in 67% of estimates; this very fact could be explained by the use of actual BW, however, when we performed the analysis excluding measurements during fluid overload, the same equation continued to overestimate the REE of patients. In accordance with this finding, a recent study on critically ill elderly patients also showed that estimate EE based only on the relationship of Kcal per Kg of BW likely overestimated EE [38]. Thus, despite being a very simple way to predict EE, this approach is likely to increase the risk of overfeeding in ICU patients with AKI.

Unlike previous equations, the Faisy-Fagon [12] and Penn-State [13] equations were developed from EE measured in mechanically ventilated critically ill patients, and dynamic variables, such as body temperature and minute ventilation that may better reflect the metabolic state of the patient were included in the predictive formulas. In our study, the equations specific for ventilated patients had different performances. The Faisy-Fagon equation was the least precise, with 64% of estimates on the overfeeding category, while Penn-State had 40% of estimates between 90–110% of values obtained by IC. In a recent study on AKI patients that used the admission BW for the calculations, those equations underestimated EE [16], again underscoring the importance of the reference BW chosen for estimation. Although the Penn-State was more accurate in our patients, it is not precise enough and its wide limits of agreement highlight the potential to under or overfeed individual patients (Figure 1). Therefore, it cannot be recommended for mechanically ventilated critically ill patients with AKI. A major problem is that no consensus exist on which BW should be utilized for EE estimation, thus explaining at least in part the differences observed in our study between measured and calculated needs, and also the dysomogeneity found among different studies. In our study, the actual BW after RRT was used; however, in some cases the patient’s weight at admission was used due to lack of BW availability during ICU stay. Nevertheless, also the analysis of the IC measurements that occurred in days with no fluid overload did not change our results. A very recent study on 205 critically ill patients compared REE measured by IC with Harris-Benedict equation calculated with 3 different reference weights (actual, ideal and predicted BW) [39]. The use of actual BW resulted in the most accurate predicted REE; however, the limits of agreement between the equation and IC measurements were very wide [39].

Finally, whether only one IC measurement at the beginning of recovery is enough to tailor nutritional prescriptions during ICU stay is still an open question. In our study there was no difference between energy measurements performed at the beginning of ICU stay and within one week, nor within 48 h, despite a vast majority of patients (68%) presenting with differences greater than ±10%, which could be clinically relevant. A retrospective study on 1171 critically ill patients found a significant (*p* < 0.0001) between-day difference, however the difference lost significance after excluding the first 2 days of hospitalization [40]. An expert position paper on IC in critically ill patients [9] states that energy expenditure of critically ill patients is very dynamic and depends on the phase and the severity of illness, treatment and extended bedrest. The same concept holds true for AKI patients [23,24]. Thus, it is recommended that, whenever the clinical condition of the patient is changing, IC should be repeated.

In our study, the formulas used for the estimation of protein needs had different performances in patients on RRT and in patients not on RRT. Because patients undergoing RRT are likely to have more fluid overload, and since BW at the end of RRT was used for the calculations, protein catabolic rate values in these patients were lower than the estimated ones. However, excluding measurements obtained during fluid overload, the results were unchanged (all patients, patients not needing RRT, and patients undergoing RRT). Normalized PCR of 1.5–2.1 g/kg/day have been obtained by the urea kinetic method in small groups of patients with AKI on different modalities of RRT [10,17,18,19,20,21]. However, different BW were considered for protein intake normalization in these studies. In two studies the lowest BW measured during ICU stay was used [19,21], in another the actual BW [20], and in the remaining 3 studies no information was available on which was the reference BW [10,17,18]. Even Guidelines not always clearly specify which BW should be considered for protein needs estimation in critically ill patients with AKI (23–24). In the present study, actual BW was preferably used, and it was the same weight used for the EE estimation. Thus, the use of different BW could explain most of the differences in protein needs estimation, underlying the importance to obtain PCR in critically ill patients. As a matter of fact, recent studies suggest that adequate protein intake correlates to better outcomes in critically ill [26,41,42,43] and AKI patients [21]; in addition, negative nitrogen balance was identified as a predictor of death in AKI patients [44].

The present study has some limitations. First of all, the sample size was relatively small, however the number of measurements performed was high, which increased the accuracy of the Bland-Altman analysis. Second, the reference BW used to estimate REE and protein needs was the actual BW. The use of the actual BW might have influenced the calculated protein and energy needs since AKI patients frequently suffer from fluid overload, however different studies used different BW, and there is no clear recommendation regarding which BW should be considered when prescribing artificial nutrition in critically ill patients with AKI. Third, the RRT modality that was used more frequently was SLED (84% of the computed treatments). In our unit, SLED protocol is based on trisodium citrate as anticoagulant [45]. It is well known that the use of trisodium citrate can provide hidden non nutritional calories (NNC) that should be considered during artificial nutrition prescription, in this study we did not consider the amount of calories provided by citrate during SLED. However, in a previous study published by our group that investigated the safety of citrate as anticoagulant for SLED, the average amount of NNC provided with our protocol was 90 kcal/SLED session of 8 h [45]. Probably, the statistical significance of the difference between received and measured energy needs would have been lost for the RRT subgroup if we had considered the NNC from citrate anticoagulation during SLED. One last consideration that should be made regarding the limitations of our study is that we did not measure the amount of amino acids lost during RRT to be considered in the actual protein needs in addition to the PCR. A recent study on five patients undergoing SLED identified a median loss of 15.7 g of amino acids per treatment [46]. This information in our study would probably not change the statistical significance of the difference between actual protein needs and estimated protein needs, and would increase the gap between actually received and protein need, only reinforcing the need for accurate protein need determination and prescription in the clinical practice.

## 5. Conclusions

In conclusion, the use of predictive equations and formulas in order to guide the nutritional approach is not accurate in critically ill patients with AKI in the ICU and should be discouraged. Whenever possible, critically ill patients with AKI should have their feeding regimen tailored by actual measurements of energy and protein needs.

## Figures and Tables

**Table 1:** Demographic and clinical characteristics (*n* = 42).

At ICU Admission	
**Age (SD)**	67 (15)
**Male sex (%)**	31/42 (72)
**Body weight at admission (kg) (SD)**	83 (25.9)
**BMI (SD)**	29 (9.4)
**APACHE II (SD)**	22.4 (7.0)
**SOFA score (median (range))**	
- Circulatory	3.0 (0–4)
- Coagulation	1.0 (0–3)
- Liver	0.0 (0–3)
- Renal	2.0 (0–4)
- Neurologic	0.0 (0–4)
- Respiratory	3.0 (0–4)
- Total	9.1 (3.2)
**Source of admission (%)**	
- Other hospital	1/42 (2)
- Medical ward	11/42 (26)
- Surgical ward	15/42 (36)
- Other ICUs	7/42 (17)
- Emergency room	8/42 (19)
**ICU admission main diagnosis (%)**	
- Trauma	3/42 (7)
- Septic Shock	12/42 (29)
- Respiratory	7/42 (17)
- Oncological	1/42 (2.25)
- Cardiac	1/42 (2.25)
- Intoxication	1/42 (2.25)
- Renal	5/42 (12)
- Vascular	6/42 (14)
- Gastrointestinal	5/42 (12)
- Other	1/42 (2.25)
**Admission type (%)**	
- Elective surgery	3/42 (7)
- Urgent surgery	19/42 (45)
- Medical	20/42 (48)
**Chronic comorbidities (%)**	
- Hypertension	31/42 (74)
- Diabetes	16/42 (38)
- COPD	6/42 (14)
- CAD	6/42 (14)
- CHF	7/42 (17)
- Peripheral vascular disease	6/42 (14)
- Immunocompromised	4/42 (10)
- Liver disease	5/42 (12)
- Malignancy	4/42 (10)
- CKD (no dialysis)	7/42 (17)
**Acute comorbidities % (at admission or up to 24 h from admission)**	
- Severe sepsis/septic shock	16/42 (38)
- IMV	31/42 (71)
- NIMV	1/42 (2)
- Shock	8/42 (19)
- AKI	31/42 (71)
- Oliguria	18/42 (40)
- RRT	6/42 (14)
- ARDS	11/42 (26)
- Vasoactive drugs	20/42 (48)
- Major bleeding (≥3 units blood needed)	7/42 (17)
**AKI characteristics**	
AKI at admission (%)	22/42 (52)
Time to AKI from ICU admission (days) (median (range))	1.0 (0–26)
sCr mg/dL at admission (median (range))	1.7 (0.4–17.6)
BUN at admission mg/dL (SD)	44 (36)
sCr mg/dL at AKI diagnosis (median (range))	2.1 (0.8–17.6)
BUN at AKI diagnosis mg/dL (SD)	52 (34)
Urinary output at AKI diagnosis (mL) (median (range))	1160 (0–4860)
APACHE II at AKI diagnosis (SD)	22.7 (6.1)
sCr mg/dL at RRT start (median (range))	4.4 (1.1–17.6)
BUN at RRT start diagnosis mg/dL (SD)	93 (52)
Urinary output at RRT start (mL) (median (range))	375 (0–4330)
APACHE II at RRT start (SD)	25.8 (5.2)
Patients on RRT (%)	19/42 (45)
Number of RRT sessions in the last 24 h before IC	38/130
Type of RRT (%)	
- SLED	32/38 (84)
- HD	4/38 (11)
- HDF	1/38 (2.5)
- CVVH	1/38 (2.5)
Duration RRT days (median (range))	9 (1–86)
Still on RRT at discharge/death	6/19 (31.6)

AKI, Acute kidney injury; ARDS, Acute respiratory distress syndrome; BMI, body mass index; BUN, Blood urea nitrogen; CAD, Chronic artery disease; CHF, Chronic heart failure; CKD, Chronic kidney disease; COPD, Chronic obstructive pulmonary disease; ICU, Intensive care unit; IMV, Invasive mechanical ventilation; NIMV, Non-invasive mechanical ventilation; RRT, Renal replacement therapy; sCr, Serum creatinine; SD, Standard deviation.

**Table 2:** Clinical characteristic on the day of IC measurement (*n* = 130 measurements).

Clinical Variable	
Maximum Temperature on the Last 24 h (°C) (Mean (SD))	37 (0.9)
Vasoactive drugs (%)	43/130 (33)
- Norepinephrine	43/43 (100)
- Epinephrine	0/43
- Dopamine	0/43
- Dobutamine	2/43 (5)
Other drugs (%)	74/130 (57)
- Barbiturates	1/74 (1)
- Propofol	6/74 (8)
- Benzodiazepines	17/74 (23)
- Neuromuscular blockers	2/74 (3)
- Morphine/Fentanyl	69/74 (93)
Insulin (%)	103/130 (79%)
Insulin IU in the last 24 h (mean (SD))	55 (36)
Blood glucose at the time of measurement (mean (SD))	138 (40)

IC, Indirect calorimetry; SD, Standard deviation.

**Table 3:** Artificial nutrition characteristics.

Variables	
**Total Days of Nutrition**	654
**EN only (%)**	294/654 (45)
**PN only (%)**	204/649 (31)
**EN + PN (%)**	156/649 (24)
**Prescribed energy and protein needs (*n* = 654)**	
- kcal/day	1551 (644)
- Protein total g/day	70.5 (38.2)
**Delivered energy and protein needs (*n* = 654)**	
- kcal/day	1408 (651) *
- Protein total g/day	63.4 (35.3) *
**Propofol (%)**	32/649 (5)
Energy received through somministration of propofol—kcal	139 (114)
**Prokinetic drugs (%)**	10/42 (24)
- Domperidone (%)	8/10 (80)
- Metoclopramide (%)	4/10 (40)

* *p* < 0.0001 vs. prescribed energy and protein. Data as mean ± standard deviation (SD). AN: Artificial nutrition; EN: Enteral nutrition; IC: Indirect calorimetry; IMV: Invasive mechanical ventilation; PN: Parenteral nutrition; RRT: Renal replacement therapy.

**Table 4:** Comparison between measured, estimated, prescribed and delivered calories and proteins.

	All Patients (*n* IC = 130; *n* PCR = 123)	On MV (*n* IC = 113)	All on RRT (*n* IC = 38; *n* PCR = 39)	RRT-MV (*n* IC = 31)	No RRT (*n* IC = 92; *n* PCR = 84)	No RRT+ MV (*n* IC = 83)
**IC**	1724 (431)	1771 (431)	1730 (445)	1798 (469)	1722 (428)	1762 (420)
**Equations**						
- Harris-Benedict with stress factor	2057 (436) ^a^	NA	2002 (277) ^a^	NA	2079 (286) ^c^	NA
- Harris-Benedict	1582 (335) ^a^	NA	1540 (213) ^c^	NA	1599 (374) ^a^	NA
- 25 kcal/kg	2116 (560) ^a^	NA	2174 (463) ^a^	NA	2092 (596) ^a^	NA
- Faisy-Fagon	NA	2089 (280) ^a^	NA	2119 (180) ^a^	NA	2079 (307) ^a^
- Penn State	NA	1793 (392)	NA	1866 (271)	NA	1796 (427)
**Prescribed**	1575 (672) ^a^	1618 (682) ^b^	1593 (561)	1659 (552)	1568 (716) ^b^	1603 (724) ^b^
**Received**	1439 (680) ^b^	1475 (690) ^a^	1443 (568) ^c^	1470 (580) ^c^	1438 (724) ^c^	1476 (727) ^c^
**ANOVA**	<0.0001	<0.0001	<0.0001	<0.0001	<0.0001	<0.0001
**PCR g/24 h**	102.1 (38.7)	NA	89.5 (29.3)	NA	107.9 (41.1)	NA
nPCR (g/kg) *	1.2 (1.09, 0.4–2.7)	NA	1.0 (0.8, 0.4–2.3)	NA	1.3 (1.2, 0.6–2.7)	NA
Calculated protein need *	108.3 (46.2)	NA	154.0 (51.8) ^d^	NA	87.1 (25.7) ^d^	NA
Prescribed protein *	76.5 (37.3) ^d^	NA	83.7 (30.1)	NA	73.2 (40.0) ^d^	NA
Received protein *	69.2 (35.3) ^d^	NA	76.7 (31.0) ^e^	NA	66.2 (37.0) ^d^	NA
**ANOVA**	<0.0001	NA	<0.0001	NA	<0.0001	NA

^a^ *p* < 0.0001 vs. IC; ^b^ *p* < 0.05 vs. IC; ^c^ *p* < 0.01 vs. IC; ^d^ *p* < 0.0001 vs. PCR; ^e^ *p* = 0.0222 vs. PCR RRT; * G of protein/24 h. Data expressed as mean ± SD or mean (median, range) in the case of nPCR. IC, indirect calorimetry; MV, mechanical ventilation; NA, non applicable; RRT, renal replacement therapy. nPCR, normalized protein catabolic rate; PCR, protein catabolic rate.

**Table 5:** Number (%) of energy expenditure estimates (calculated using the different equations) within 90% and 110% of IC values, and number (%) of estimates that would result in under (<90% IC) or overfeeding (>110% IC).

Equation	% of Estimates 90–110% of IC			% of Estimates <90% of IC (Underfeeding)			% of Estimates >110% of IC (Overfeeding)		
	All Patients	RRT	No RRT	All Patients	RRT	No RRT	All Patients	RRT	No RRT
**HB + 30% SF**	26	18	29	5	8	3	9	74	67
**HB**	38	42	36	46	47	46	16	11	18
**25 kcal/kg/day**	28	18	32	5	3	7	67	79	62
**Faisy-Fagon**	31	31	31	5	7	5	64	62	64
**Penn-State**	40	38	40	27	24	27	34	38	32

IC, indirect calorimetry; HB, Harris-Benedict equation; SF, stress factor. Faisy-Fagon and Penn-State are only for patients on mechanical ventilation.

**Table 6:** Comparison between measured and estimated REE of patients not on fluid overload on the day of IC measurement.

	All Patients (*n* = 81)	On MV (*n* = 72)
**IC**	1735 (413)	1791 (399)
**Equations**		
Harris-Benedict with stress factor	1995 (462) ^a^	NA
Harris-Benedict	1535 (356) ^a^	NA
25 kcal/kg	2031 (576) ^a^	NA
Faisy-Fagon	NA	2081 (298) ^a^
Penn State	NA	1767 (427)
**ANOVA**	<0.0001	<0.0001

^a^ *p* < 0.0001 vs. IC; Data expressed as mean ± SD. IC, indirect calorimetry; MV, mechanical ventilation.

## Supplementary Materials

The following are available online at www.mdpi.com/ 2072-6643/9/8/802/s1, Table S1: Equations used for the estimation of resting energy expenditure.
