# Supplementary material for: Energy and Protein in Critically Ill Patients with AKI: A Prospective, Multicenter Observational Study Using Indirect Calorimetry and Protein Catabolic Rate
Source: Nutrients. 2017 Jul 26;9(8):802. doi: 10.3390/nu9080802 (PMC5579596; doi:10.3390/nu9080802)
Supplement: Supplementary file 1 [file nutrients-09-00802-s001.zip › nutrients-206092-supplementary.pdf]

Supplementary material

Table S1. Equations used for the estimation of resting energy expenditure.

ESPEN guideline [23,24]	25 Kcal/Kg
Harris-Benedict [25]	REE (male) = 66.45 + 13.75 x body weight + 5 x height - 6.76 x age<br>REE (female) = 655.1 + 6.56 x body weight + 1.85 x height - 4.65 x age
Penn-State [13]	(REE male or female)[a] x 0.85 + 175 x Tmax + 33 x Ve – 6433
Faisy-Fagon [12]	8 x body weight + 14 x height + 32 x Ve + 94 x Tmax – 4834

[a] REE is resting energy expenditure calculated using Harris-Benedict equation for male or female

In all equations body weight is in Kg, height is in centimeters.

Tmax is the maximum temperature in the last 24h in degrees Celsius, Ve is minute ventilation in liters/minute.
